# The Frequency of MEFV Gene Mutations and Genotypes in Sanliurfa Province, South-Eastern Region of Turkey, after the Syrian Civil War by Using Next Generation Sequencing and Report of a Novel Exon 4 Mutation (I423T)

**DOI:** 10.3390/jcm7050105

**Published:** 2018-05-07

**Authors:** Evren Gumus

**Affiliations:** Department of Medical Genetics, Faculty of Medicine, University of Harran, 63000 Sanliurfa, Turkey; evreng@ymail.com

**Keywords:** MEFV mutations, genetics, rheumatology, FMF, next generation sequencing

## Abstract

Background: Familial Mediterranean Fever (FMF) is a genetic disorder characterized by recurrent episodes of fever and abdominal pain. Mutations in the Mediterranean fever (MEFV) gene are localized on the p arm of chromosome 16. Over 333 MEFV sequence variants have been identified so far in FMF patients, which occur mostly in the 2nd and 10th exons of the gene. Methods: In this study, 296 unrelated patients with clinical suspicion of FMF, which were admitted during January–December 2017, were retrospectively reviewed to identify the frequency of MEFV gene mutations by using next generation sequencing. Results: Eighteen different mutations, 45 different genotypes and a novel exon 4 (I423T) mutation were identified in this study. This mutation is the fourth mutation identified in exon 4.The most frequent mutation was R202Q, followed by M694V, E148Q, M680I, R761H, V726A and R354W. Conclusions: One of the most important aims of this study is to investigate the MEFV mutation type and genotype of migrants coming to Sanliurfa after the civil war of Syria. This study also examines the effect of the condition on the region’s gene pool and the distribution of different types of mutations. Our results indicated that MEFV mutations are highly heterogeneous in our patient population, which is consistent with the findings of other studies in our region. Previously used methods, such as Restriction Fragment Length Polymorphism (RFLP), do not define uncommon or especially novel mutations. Therefore, Next Generation Sequencing (NGS) analysis of the MEFV gene could be useful for finding novel mutations, except for those located on exon 2 and 10.

## 1. Introduction

Familial Mediterranean fever (FMF, #MIM 249100) is an autosomal recessive inherited auto-inflammatory disease characterized by fever, arthritis, peritonitis, pleuritis, myalgia, splenomegaly, erysipelas-like skin lesions and renal amyloidosis [1]. The most frequent symptoms of this disease are abdominal pain and fever. Although this is an autosomal recessive disease, heterozygous individuals have also been shown to exhibit classical disease-related symptoms [2]. This disease is primarily observed in Mediterranean countries, such as Turkey, Syria, Armenia, Tunisia, Morocco, Israel and Islamic Republic of Iran [3]. The calculated prevalence of FMF is about 1/1000, while the carrier ratio is 1/5 in Turkey [4]. The disease onset is usually during the first decade of life. The Mediterranean fever (MEFV) gene is located on chromosome 16 and is consisted of ten exons. The MEFV gene codes for a 781 amino acid protein named pyrin (or marenostrin), which regulates inflammation and apoptosis. Pyrin is primarily expressed in monocytes and neutrophils [3,5]. Over time, more than 100 disease-causing mutations and more than 330 sequence variants (mutations and polymorphisms) have been determined in the MEFV gene. The majority of these variants are in exon 2 and 10, especially in the PRYSPRY domain, which is responsible for protein–protein interactions in exon 10 [6,7]. The four missense mutations in exon 10 (M680I, M694V, M694I, V726A) comprise about 75–85% of the MEFV gene mutations in the Mediterranean region [3,8].

Sanliurfa was originally not a center for migration but after the Syrian civil war, Syrian citizens constitute one-fourth of the city’s entire population. Recently, MEFV gene mutation spectrum studies have been conducted in the various districts of Turkey and south-eastern Anatolia. However, none of these studies were conducted with Next Generation Sequencing (NGS). In this retrospective study, 296 unrelated patients with a clinical diagnosis of FMF were retrospectively analyzed to identify the frequency of MEFV gene mutations. In addition, a novel exon 4 missense mutation (I423T) of the MEFV gene was identified. We aimed to: (i) determine the frequency of MEFV gene mutations in the Sanliurfa province of south-eastern Anatolia; (ii) compare the results of the MEFV mutations spectrum before and after the migration of the Syrian refugees to the area; (iii) compare the results with other geographic regions of Turkey; (iv) compare the results with Turkish and Syrian patients; (v) analyze the country overall; and (vi) discuss the use of NGS in FMF patients..

## 2. Material and Methods

Overall, 296 unrelated patients (76 Syrian, 220 Turkish) from different clinics, especially both pediatric rheumatology and adult rheumatology clinics, were selected as they had clinically suspected FMF according to the Tel-Hashomer criteria. These patients were retrospectively investigated between 1 January 2017 and 31 December 2017. All Turkish and Syrian patients were from the Sanliurfa province in the south-eastern region of Turkey. The study was approved by the Institutional Review Board of Faculty of Medicine in Harran University (Ethic Committee Document Number: 04.01.2018/01-12) and the study was conducted in accordance with the Declaration of Helsinki. Complete blood samples were taken from the antecubital vein via vacutainer tubes containing ethylenediaminetetraacetic acid (EDTA) (BD, Franklin Lakes, NJ, USA). DNA was isolated from blood samples using Magpurix Blood DNA Extraction Kit 200 (Zinexts LSC, New Taipei City, Taiwan (R.O.C.)). The quantitative purity determinations and fluorometry analysis were performed. PCR was performed in the BIORAD T100 Thermal Cycler (Bio-Rad, Dubai, United Arab Emirates) using the NEXTflex Mediterranean Fever Amplicon Panel (Bioo Scientific, Austin, TX, USA). This panel contains a total of 24 primer pairs in two pools, which allows for the amplification and sequencing of all coding exons of the MEFV gene. For NGS, PCR products were loaded to MiniSeq (Illumina, San Diego, CA, USA) according to the manufacturer’s protocol. All data were analyzed in the Genomize bioinformatic tool (SEQ, Istanbul, Turkey). Statistical data were analyzed using SPSS 23.0 (IBM SPSS, Chicago, IL, USA) program. The ratio *t*-test was used for the comparison of data. For all tests, *p* < 0.05 was considered as significant. 

## 3. Results

**Figure 1:**
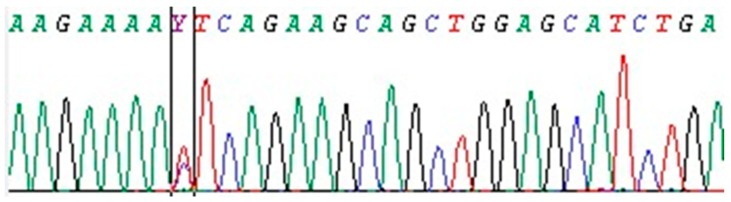
Sequence electropherogram of the novel I423T mutation. The two lines indicates T > C substitution at the nucleotide position c.1268 in exon 4 of the MEFV gene.

Among the 296 patients, 150 (50.7%) were females and 146 (49.3%) were males. The mean age of patients was 20.4 ± 4.95 years. According to our results, 45.6% (135/296) of the patients had mutations in MEFV gene, while 54.4% (161/296) of the patients had no mutations. Of the 135 individuals with a mutation, 35 were Syrian and 100 were Turkish. Out of 135 patients with mutations, 64 (47%) were female and 71 (53%) were male. There were no significant differences in the prevalence of MEFV mutation between males and females (*p* = 0.3). The patients were classified into four groups according to allele status: heterozygous, homozygous, compound heterozygous and complex genotype. No significant differences between genders were detected for heterozygous, homozygous, compound heterozygous and complex type patients. Of those 135 patients with mutations, 50 (37%) were heterozygous, 16 (12%) were homozygous, 62 (46%) were compound heterozygous and 7 (5%) were complex genotype. The most common mutation according to allelic frequency was R202Q, followed by M694V, E148Q, M680I, R761H, V726A, R354W, M694I, A744S, R408Q, R369S, I591T, T267I, E148V, V487L, L110P, P188P and P124P (Table 1). The most common heterozygous mutation was E148Q, followed by R202Q, M694V, R761H, A744S, M680I, M694I, V726A, T267I, R354W and V487L. The most common homozygous mutation was R202Q, followed by M680I, R761H, M694V, E148Q and V726A (Table 2). The three most common compound heterozygous mutations were M694V/R202Q, R354W/E148Q and I591T/P369S (Table 3). Six different complex genotypes were detected in a total of seven patients (Table 4). Exon 2 was affected in 87 of 135 (64.4%) patients with a mutation. This ratio was 8.1% for exon 3 and 63% for exon 10. No homozygous mutation was observed in exon 3. We identified 18 different known mutations (7/18 with exon 2, 6/18 with exon 10, 3/18 with exon 3, 1/18 with exon 5 and 1/18 with exon 9) and a novel exon 4 mutation (I423T).The exon 4 missense mutation is caused by the substitution of an isoleucine with a threonine as a result of a T to C transition at the nucleotide position 1268 in exon 4 of the MEFV gene. For the in-silico prediction tools, this variant is disease causing (PolyPhen: probably damaging; Sorting Tolerant From Intolerant (SIFT): deleterious; Provean: deleterious; and Mutation Taster: disease causing). This mutation was found in heterozygous form (Figure 1). This patient, who was a 12-year-old girl, had recurrent episodes of fever and abdominal pain over the previous four years, which responded to colchicine treatment. With this novel mutation, we identified 19 different mutations and 45 different genotypes (12 heterozygous, 6 homozygous, 21 compound, 6 complex). The total number of Syrian patients was 76 (40 female, 36 male). According to our analysis, 46% (35/76) of the Syrian patients had mutations in the MEFV gene. The genotype distribution of the Syrian patients included 12 heterozygous, 3 homozygous, 15 compound heterozygous and 5 complex (Table 5). There was no significant difference between the Syrian and Turkish patients in terms of: (i) gender distribution; (ii) average age; (iii) allele frequency and (iv) genotype (*p* > 0.05). However, there was a significant difference in the mutation type (complex) (*p* = 0.032).

## 4. Discussion

The study was performed in a university hospital in south-east Turkey by using NGS. This is the first time that people with FMF mutation types in this region were analyzed and reported using NGS. FMF affect both genders equally However, female or male dominance has also been reported [3]. Our results showed a slight male predominance (1.1/1). We identified one novel mutation (I423T) and many rare mutations in the MEFV gene. This novel variant was neither found in ExAC nor 1000G and was disease-causing according to the mutation taster data. This missense mutation resulting in the replacement of non-polar isoleucine with polar threonine may produce a conformational change in the protein construction and reduce protein function. Exon 4 and 7 mutations are the least frequently observed mutations in the MEFV gene. This mutation is the fourth mutation identified in exon 4.

We detected no mutations in 161 (54.4%) of the 296 patients in our study. In other studies from Turkey, the prevalence of patients without mutations were in the range of 38.2–65.9% [8,9,10,11].

In regional and country-wide studies, the six most frequently observed mutations are R202Q, M694V, E148Q, M680I, V726A and M694I [4,8,11,12]. In this study, the six most frequently observed mutations were R202Q, M694V, E148Q, M680I, R761H and V726A. The most remarkable genotype is M694V/R202Q, which had higher rates (15 patients) compared to the other genotypes.

R202Q was found in 5–34% of the Turkish population. Our result was 24%. The R202Q frequency in Syrian patients was 23% (15/65), while this was 24.6% (44/179) in Turkish patients. No statistical difference was observed between the two groups. In some papers, it is still listed as a polymorphism. However, it is accepted as a mutation or regulatory element in other studies. Some of the studies emphasize that homozygous or compound heterozygous R202Q mutation types could be the cause of FMF and amyloidosis [13,14,15]. Moreover, it has been suggested that the patients with homozygous R202Q alterations or with compound heterozygous R202Q mutations may require colchicine treatment [16]. Overall, these results suggest that R202Q is a disease-causing mutation. In our study, E148Q was the third most common mutation with a frequency of 16%. The majority of E148Q mutations were found in the compound heterozygous form. E148Q is one of the five most common mutations and it is the first or second most frequent mutation in many studies from Turkey [8,9].

It is important to consider the method used for mutation detection. If we had used the 12-mutation assay panel (E148Q, P369S, F479L, M680I (G/C), M680I (G/A), I692del, M694V, M694I, K695R, V726A, A744S and R761H) instead of NGS, we would not be able to see the changes observed in 1/3 of the patients. If we exclude the R202Q mutation, this ratio would be 13%. This ratio would be 8.2% if we were only sequencing exon 2 and 10. If the exons 2, 3 and 10 were sequenced, this ratio would be only 1.45%. However, except for NGS, a novelI423T mutation in exon 4 could not be detected by other methods. In the context of these data, we think that the sequencing of exons 2, 3 and 10 is the most appropriate option in our routine medical genetic polyclinic, because NGS is a high-cost analysis method.

Four years ago, Abuhandan et al. investigated 186 patients with FMF in our city before the Syrian civil war and the most frequent mutations were R202Q (33.3%), M694V (22.6%), E148Q (22%), V726A (7.5%), R761H (4.3%), M680I (3.8%) and others (6.5%). Although our study and the study by Abuhandan et al. showed that the six most common mutations were similar, the rate of other mutations in our study was 14%. Abuhandan et al. found that the compound heterozygous frequency was 10.8%, although this rate was 46% in our study. This can be explained by the sample size and NGS. Abuhandan et al. included no complex type [17], with had a frequency of5.2% in our study. This can be explained by NGS, sample size and perhaps the most important thing, the timing of this study as it was performed after the migration.

FMF is common in Syrians and the carrier rate is similar to the Turkish population (17.5%) [18]. In the Syrian FMF patients, the most frequent mutation was M694V, followed by V726A, E148Q and M680I [19]. In our study, we can conclude that the distribution in the Syrian patient group is similar except for the V726A mutation. This can be explained by the small number of Syrian patients. In our country, the complex type ratio varies between 0.7% and 1.3% [20,21]. This rate was 2% in the Turkish patient group of our study, which is similar to the previous studies. This rate was 6.7% in another study conducted in Syria [19]. The complex genotype rate was 14% in the Syrian patient population of our study. This rate was 5.2% in our study group. These results provide a good example of the variation of gene frequency and genotype distributions in the immigrant communities. We believe that if we had more Syrian patients and a bigger sample (>1000 patients), we could see this effect on the mutation frequency.

## 5. Conclusions

Our study revealed different results from the previous results of Turkey in general. The reason might be that the analysis of all of our participants was conducted with NGS and with a different sample size. Furthermore, our patients were from Sanliurfa, which has been a key immigration destination in Turkey after the Syrian civil war. The NGS analysis of the MEFV gene could be useful for the detection of every mutation type (novel, rare, known) in regions where FMF is common and has mutational diversity.

## Figures and Tables

**Table 1:** Allele frequencies of MEFV mutations among 135 patients.

Mutation Type (Allele)	Number of Alleles	Allele Frequency (%)
R202Q	59	24
M694V	42	17
E148Q	40	16
M680I	24	10
R761H	20	8
V726A	16	6.5
R354W	8	3.3
M694I	7	2.8
A744S	6	2.5
R408Q	6	2.5
P369S	3	2.5
I591T	2	1.2
T267I	1	0.8
E148V	1	0.4
V487L	1	0.4
L110P	1	0.4
P283L	1	0.4
E230K	1	0.4
Total	244	100

**Table 2:** Genotype distribution of heterozygous and homozygous patients.

Genotype	Heterozygous (*n* = 50)	Homozygous (*n* = 16)
E148Q	12 (24%)	1 (6.2%)
R202Q	9 (18%)	6 (37.5%)
M694V	8 (16%)	2 (12.5%)
R761H	7 (14%)	2 (12.5)
A744S	4 (8%)	-
M680I	3 (6%)	4 (25%)
M694I	2 (4%)	-
V726A	1 (2%)	1 (6.2)
T267I	2 (4%)	-
R354W	1 (2%)	-
V487L	1 (2%)	-
Total	50 (100%)	16

**Table 3:** Genotype distribution of compound heterozygous patients.

Genotype	Compound Heterozygous (*n* = 62)
M694V/R202Q	15 (24%)
R354W/E148Q	6 (9%)
I591T/P369S	6 (9%)
R202Q/E148Q	4 (6%)
M694V/V726A	3 (4.8%)
E148Q/M680I	3 (4.8%)
R202Q/I591T	3 (4.8%)
V726A/M680I	3 (4.8%)
R761H/R202Q	2 (3.2%)
M680I/M694I	2 (3.2%)
M694V/E148Q	2 (3.2%)
V726A/E148Q	2 (3.2%)
R761H/M680I	2 (3.2%)
M694V/V726A	2 (3.2%)
V726A/E148V	1 (1.6%)
M680I/A744S	1 (1.6%)
A744S/R202Q	1 (1.6%)
M694V/M680I	1 (1.6%)
V761H/V726A	1 (1.6%)
R202Q/R354W	1 (1.6%)
R202Q/P283L	1 (1.6%)
Total	62 (100%)

**Table 4:** Genotype distribution of complex genotype patients.

Genotype	Complex Genotype (*n* = 7)
R202Q/R202Q/M694V/M694V ^s,t^	2
E230K/R202Q/M694V/M694V ^t^	1
M694V/M694V/E148Q ^s^	1
R202Q/R202Q/M694V ^s^	1
E148Q/E148Q/M680I ^s^	1
E148Q/E148Q/M694V/M694V ^s^	1
Total	7 (100%)

^t^: Turkish patient, ^s^: Syrian patient.

**Table 5:** Genotype distribution of heterozygous and homozygous genotypes in Syrian patients.

Genotype	Heterozygous (*n* = 12)	Homozygous (*n* = 3)
E148Q	3 (25%)	-
R202Q	3 (25%)	1
M694V	2 (8.3%)	1
R761H	1 (8.3%)	-
M680I	1 (16.5%)	1
M694I	1 (8.3%)	-
V726A	1 (8.3%)	-
Total	12 (100%)	3 (100%)
